# Enhanced Cell Osteogenic Differentiation in Alendronate Acid and Flufenamic Acid Drug-Impregnated Nanoparticles of Mesoporous Bioactive Glass Composite Calcium Phosphate Bone Cement In Vitro

**DOI:** 10.3390/ph16050680

**Published:** 2023-05-01

**Authors:** Shih-Ming Liu, Jian-Chih Chen, Ssu-Meng Huang, Shang-Hong Lin, Wen-Cheng Chen

**Affiliations:** 1 Advanced Medical Devices and Composites Laboratory, Department of Fiber and Composite Materials, Feng Chia University, Taichung 407, Taiwan; 0203home@gmail.com (S.-M.L.); d830191@yahoo.com.tw (J.-C.C.); dream161619192020@gmail.com (S.-M.H.); nel3581@gmail.com (S.-H.L.); 2 Department of Orthopedics, Faculty of Medical School, College of Medicine, Kaohsiung Medical University, Kaohsiung 807, Taiwan; 3 Department of Orthopedics, Kaohsiung Medical University Hospital, Kaohsiung 807, Taiwan; 4 Department of Fragrance and Cosmetic Science, College of Pharmacy, Kaohsiung Medical University, Kaohsiung 807, Taiwan; 5 Dental Medical Devices and Materials Research Center, College of Dental Medicine, Kaohsiung Medical University, Kaohsiung 807, Taiwan

**Keywords:** nanoparticles, mesoporous bioactive glass, calcium phosphate bone cement, composites, controlled release, drug delivery, in vitro

## Abstract

This study aims to compare the anti-osteoporotic drugs alendronic acid (ALN) and flufenamic acid (FA) alone impregnate into nanoparticles of mesoporous bioactive glass (nMBG), which further composites calcium phosphate cement (CPC) and investigates their in vitro performance. The drug release, physicochemical properties, and biocompatibility of nMBG@CPC composite bone cement are tested, and the effect of the composites on improving the proliferation and differentiation efficiency of mouse precursor osteoblasts (D1 cells) is also investigated. Drug release shows that FA impregnates nMBG@CPC composite, a large amount of FA is released rapidly within 8 h, gradually reaching a stable release within 12 h, followed by a slow and sustained release within 14 days, and then reaches a plateau within 21 days. The release phenomenon confirms that the drug-impregnated nBMG@CPC composite bone cement effectively achieves slow drug delivery. The working time and setting time of each composite are within 4–10 min and 10–20 min, respectively, meeting the operational requirements of clinical applications. The addition of nMBG nanoparticles in the CPC matrix did not prevent the aggregation phenomenon under microstructural observation, thus resulting in a decrease in the strength of the nMBG@CPC composite. However, after 24 h of immersed reaction, the strength of each 5 wt.% nMBG impregnated with different concentrations of FA and ALN is still greater than 30 MPa, which is higher than the general trabecular bone strength. The drug-impregnated nMBG@CPC composites did not hinder the product formation and exhibit biocompatibility. Based on the proliferation and mineralization of D1 cells, the combination of nMBG with abundant FA and ALN in CPC is not conducive to the proliferation of D1 cells. However, when D1 cells are contact cultured for 21 days, alkaline phosphatase (ALP) enzyme activity shows higher ALP secretion from drug-impregnated nMBG@CPC composites than drug-free composites. Accordingly, this study confirms that nMBG can effectively impregnate the anti-osteoporosis drugs FA and ALN, and enhance the mineralization ability of osteoblasts. Furthermore, drug-impregnated nMBG applications can be used alone or in combination with CPC as a new option for osteoporotic bone-filling surgery.

## 1. Introduction

With the gradual aging of the population, bone defects caused by osteoporosis have become a common clinical problem in orthopedics. In today’s aging society, osteoporosis has the highest incidence among bone diseases. Osteoporosis is primarily due to faster resorption by osteoclasts than remodeling by osteoblasts. With the advancement of medical technology, inhibiting osteoclasts has become the main method for the treatment of osteoporosis [1]. In the setting of osteoporosis, patients are prone to fractures [2,3,4]. Medication is the main method commonly used clinically to treat osteoporosis. In addition, when repairing bone defects, considerations for selecting an adjustable bone resorption substitute for implantation are more important than strength considerations. Due to the decay or degradation properties of the bone substitute itself, drug-loaded carriers are usually compounded into the bone substitute to regulate the release of the drug [5,6,7,8,9]. This strategy aims to precisely reestablish the homeostasis between osteoblasts and osteoclasts to repair bone defects.

Calcium phosphate bone cement (CPC) is the most promising and commonly used bone-filling material in the clinical treatment of bone defects [10,11,12,13]. The main ingredient is a powder mixed with a hardening solution to form an injectable slurry during delivery, which solidifies in situ after implantation to produce an apatite product similar to human bone. CPC has good plasticity and injectability before hardening, as well as good biocompatibility, osteoconductivity, and osteogenesis.

Scholars have successfully prepared mesoporous bioactive glass (MBG) by evaporation-induced self-assembly using a sol–gel method with surfactants as pore templates [14,15]. Silicate synthesis in an alkaline environment would form nano-scale MBG (nMBG) with a particle size of about 200 nm and a single spherical structure [16,17]. Supplemented by the microemulsion method, a dandelion-like radial mesoporous structure would be formed, which could greatly increase the specific surface area and the amount of drug adsorption.

According to research [18], bisphosphonates can reduce osteoclast activity and enhance osteoblast differentiation, thereby improving osteoporosis and reducing the risk of fractures. These drugs are available with and without nitrogen, and they inhibit osteoclasts in different ways. The terminal bisphosphate of non-nitrogen-containing bisphosphonate will be replaced by pyrophosphate at the terminal of ATP, forming a non-hydrolyzable ATP-like compound in the cell; this compound cannot metabolize the energy of the cell and then induces osteoclast apoptosis. Nitrogen-containing bisphosphonates inhibit osteoclasts by inhibiting protein prenylation, thereby inhibiting nickel pyrophosphate synthase (FPPS) in the HMG-CoA pathway and blocking farnesol and geranylgeraniol metabolites. Ultimately, they inhibit the prenylation of proteins and reduce the prenylation of lipids, so functional proteins cannot be fixed on the cell membrane of osteoclasts, leading to the apoptosis of osteoclasts. Alendronic acid (ALN), as the most widely used nitrogen-containing bisphosphonate in the market, is relatively mature in development. In this experiment, ALN was selected to compare the curative effect with flufenamic acid (FA), a relatively new anti-osteoporosis drug.

FA is a derivative drug of fenamic acid and a kind of non-steroidal anti-inflammatory analgesic drug (NSAIDs). NSAIDs are often used to relieve pain in patients with osteoporosis. In the past few decades, menopausal women who have not received estrogen replacement tend to have increased bone density after taking NSAIDs. In addition to promoting osteoblastic activation, FA will reduce the activity of MAPK subfamily P38 and ERK by macrophages through the RANKL/MAPKs pathway and inhibit the phosphorylation of the MAPKs pathway, thereby inhibiting osteoclast differentiation and reducing its number [19].

MBG has the following four forms of the adsorbed drug: Type I, where the drug is adsorbed on the side of the mesopore; Type II, where the drug is physically embedded in the mesopore; Type III, where the hydroxyl and amino groups of the drug are combined with Si-OH and P-OH inside or on the surface of MBG to form hydrogen bonds; Type IV, where drugs are physically adsorbed outside the mesopore [20,21,22]. In the Fickian diffusion mechanism, drug molecules on the MBG surface and outside the mesopores (type I and IV) are released first at an early stage, resulting in a fast release. Then, in the next stage, the drug molecules are either inside the MBG mesopores or connected by hydrogen bonding (type II and III), leading to a slow release of the drug [23].

In this study, ALN in bisphosphonates and the emerging drug FA were individually impregnated into nMBG. The two drug-impregnated nMBGs were combined with commercialized CPC to form nBMG@CPC composites. The drug release, physicochemical properties, and biological properties of the composites, including compatibility and bone mineralization capacity, were studied.

## 2. Results and Discussion

### 2.1. Observation of FA-Impregnated nMBG

**Figure 1:**
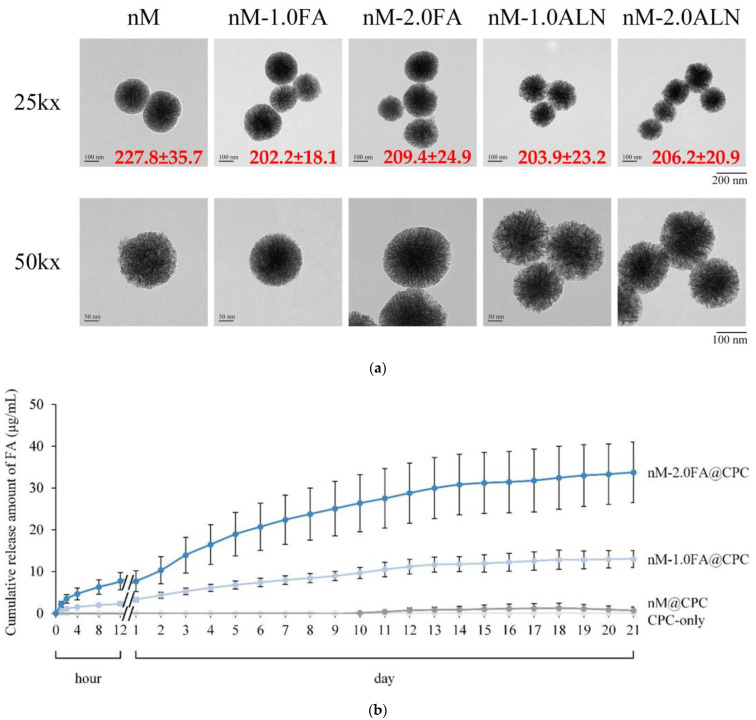
(**a**) TEM microstructure and the particle size distribution of drug-free nMBG (nM) and drug-impregnated nanoparticles (nM-1.0FA, nM-2.0FA, nM-1.0ALN, and nM-2.0ALN) in each group (*n* = 10); (**b**) Cumulative drug release of FA-impregnated nMBG@CPC composite as bone cement (*n* = 8).

Figure 1a shows that all nanoparticle populations of nMBG have a radius of about 200 nm, are spherical and have radially arranged pores. After drug impregnation in FA and ALN, sustained release of type II and type III release models in nMBG could be expected since the difference in particle distribution between different nMBG groups was not significant, implying that the drug was mainly loaded in the cavities of central scattering rather than being attached to the outside of the nMBG sphere. The slurry of the CPC composite as a drug carrier can be used for slow drug release, and its clinical application is more diverse after being loaded with drugs. The drug release mechanism conforms to the following mathematical model proposed by Korsmeyer et al. [24]:

(1) $$X_{i}=\;M_{t}{/M}_{\infty }{=k\;\times \;t}^{n}$$

where M_*t*_ represents the mass of the drug released at time *t*; M_∞_ shows the mass of the drug released as the time approaches infinity; k reveals the parameter describing all the geometric structures and characteristics of the model; *n* is Fickian diffusion model correlation coefficients [11,25].

Figure 1b shows the cumulative release profiles of drug-impregnated nMBG@CPC with different concentrations of FA. The drug release curve could be divided into several stages. In the first stage, the drug was released proportionally within 8 h, which may be due to the type I and type IV release of nMBG nanoparticles adsorbed on and off the surface after contact with liquid. From 8 h to 12 h of soaking, the release was seen to a milder level. At this time, the release was slower than the first stage. In the second stage, the release curve gradually rises, indicating that the drug impregnated in the mesopores of nMBG embedded in the CPC matrix began to be released. The drug release in the third phase was slow, and the release did not reach a plateau until day 14 due to the compact structure of the hardened CPC composite. Based on a calculation using Equation (1), the result of *n* is 0.46, which indicates that the drug release is non-Fickian or anomalous transport [11]. The mechanism of drug release is governed by diffusion and nMBG decay. The diffusion and decay rates were comparable, and the anomalous transport had a cylindrical geometry (0.45 < *n* < 0.89).

The total release concentrations of CPC@nM-1.0FA and CPC@nM-2.0FA after soaking for 21 days were 13.0 ± 2.1 and 34.1 ± 7.2 μg/mL, respectively. Nanoparticles of nMBG showed higher release rates when impregnated with higher concentrations of FA, and this release concentration can effectively inhibit the activity of osteoclasts [19]. Therefore, the concentration of another drug, ALN, in subsequent experiments was referred to as the same as that of FA.

### 2.2. Operations of CPC@nMBG Composite Bone Cement

#### 2.2.1. Working Time for Operation and Setting Time for Adhesion and Anti-Washing

**Figure 2:**
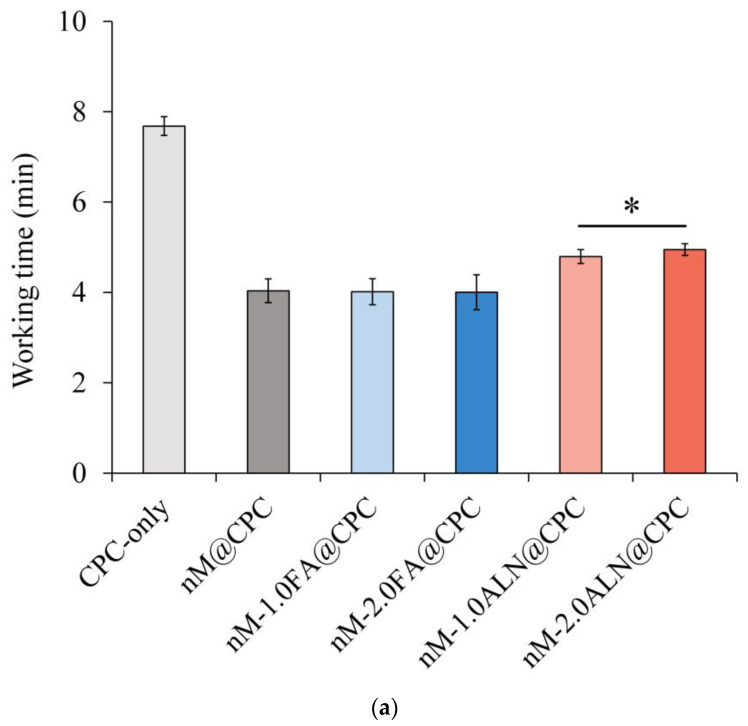

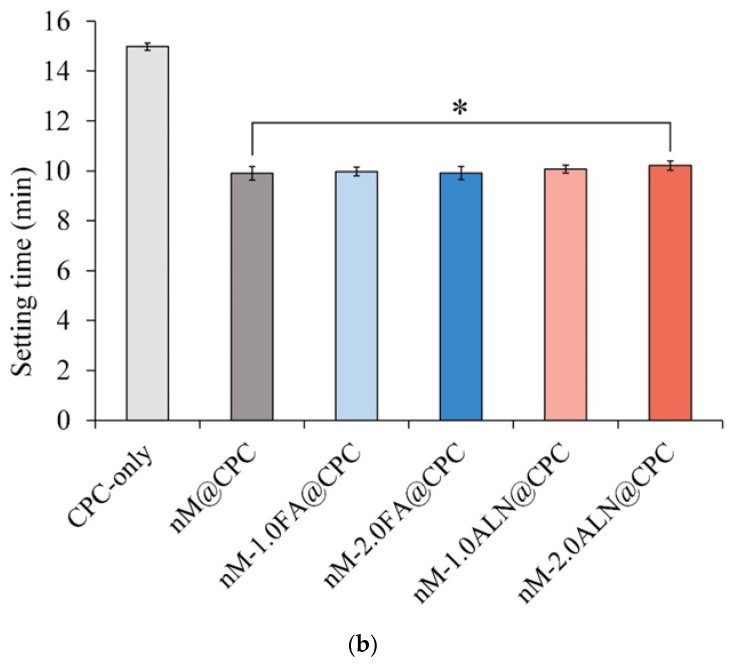
(**a**) Working time of FA- and ALN-impregnated nMBG@CPC composite bone cements compared with the control groups of CPC-only and drug-free nM@CPC; (**b**) Setting time of FA- and ALN-impregnated nMBG@CPC composites compared with the control groups of CPC-only and drug-free nM@CPC; * between-group symbols are statistically significant (*p* < 0.05).

Working time is defined as the time during which no mutual adhesion, and setting time is when the mixed slurry exhibits a certain strength after initial hardening. Figure 2a,b shows the working time and hardening time results for each group. The working time in the CPC group was about 7 min, while that of the drug-impregnated nMBG@CPC composite was shortened to about 4–5 min. The curing time in the CPC group was about 15 min, and that in the nMBG@CPC group was 10 min. In terms of injectability, the lack of viscous flow is a serious limitation of CPC. Fluidity was increased by increasing the wetting ratio but only to a limited extent [26]. For example, increasing the hardening liquid causes the cement mixture to form a loose paste, and the liquid portion is pushed out through the needle. High wetting ratios can increase the setting time to unacceptable values and can cause the washing away of the unset cement if exposed to an aqueous environment. Studies have shown that a curing time of 15–20 min is appropriate after delivering CPC slurry to the repair sites for bone defect rehabilitation [27].

The nMBG@CPC composites reduced the working and setting times, especially the working time because adding a small amount of nMBG could shorten the working time of CPC by absorbing the residual water molecules into the nMBG [28]. The working time of the ALN group was particularly longer than that of the other nMBG composite groups but shorter than that of CPC-only. In addition, the setting time of the nM-2.0ALN@CPC was the only group that significantly differed from that of the drug-free nM@CPC group because ALN has a high affinity for calcium ions, thereby prolonging the working time [29,30].

#### 2.2.2. Injectability

The nMBG@CPC composite bone cement is a muddy slurry before hardening and thus needs to be injected into the bone defect with a syringe during application. If the hardening time is too short and the viscosity is too high, then it will solidify in the syringe and cannot be injected; resistance to disintegration is poor when it disintegrates when it comes in contact with liquid after injection, thereby limiting its clinical application [31].

**Figure 3:**
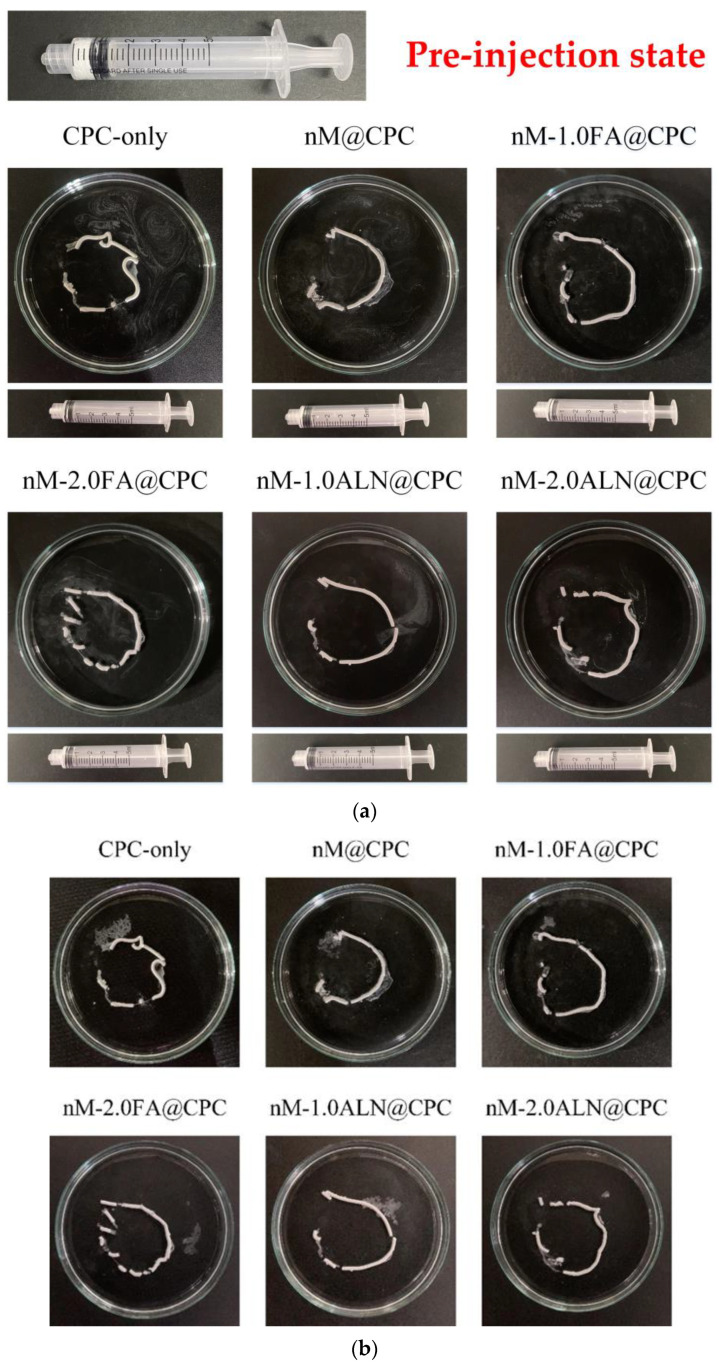
(**a**) Real-time optical images of each group of drug-impregnated nMBG@CPC composites injected in ddH_2_O within 1 min; all syringes can be pushed to the end and only a small amount of composite bone cement residue was found after injection; (**b**) optical image comparison of injected composite bone cements: FA and ALN impregnated nMBG@CPC maintained in ddH_2_O for 24 h.

Figure 3a shows a live image of the injection test of each drug-impregnated nMBG@CPC composite injected in double-distilled water (ddH_2_O) for 1 min. Each group successfully pushed the sample out of the syringe, and the residual rate of the samples in each group that could not be prevented from remaining in the syringe was about 12~15%. This indicates that nMBG@CPC composite bone cement has a good injection performance [32]. After soaking in water for 1 day (Figure 3b), the shape was well maintained, indicating that each group had good resistance to disintegration.

The main advantage of slurry–composite bone cement is that it can be delivered by syringe. Therefore, some researchers have attempted to inject CPC in paste form; that is, self-setting CPC slurry with the viscous flow for complete extrusion from the syringe [26]. One of the most notable reports is that of Constantz et al. (1995) [33], who achieved remarkable success in the minimally invasive treatment of acute radius fractures by percutaneous CPC administration.

### 2.3. Physicochemical Characterization of CPC@nMBG Composites after 24 h of Immersion

#### 2.3.1. Compressive Strength and Fracture Surface Observation

**Figure 4:**
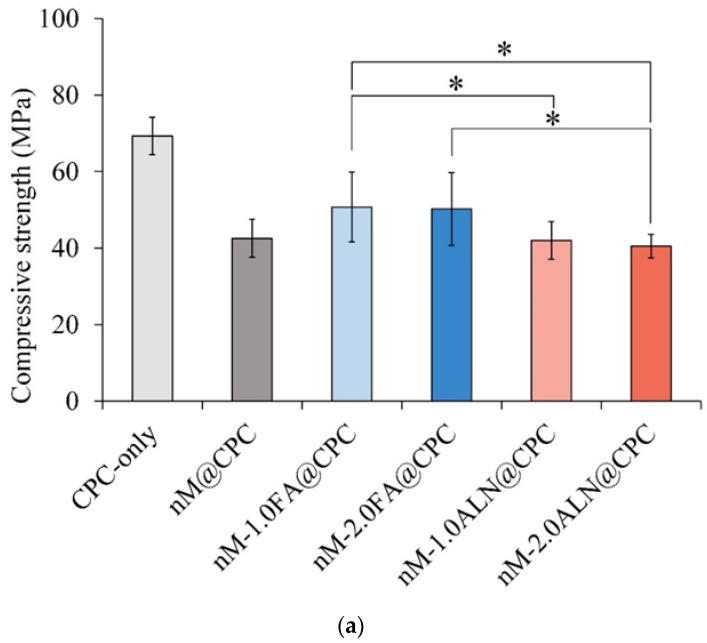

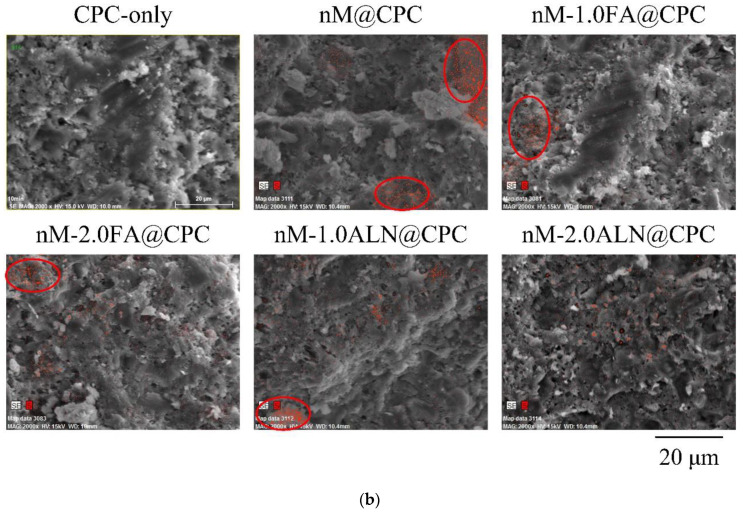
(**a**) Compressive strength of FA- and ALN-impregnated nMBG@CPC after soaking in SBF for 24 h (*n* = 10); * between-group symbols are statistically significant (*p* < 0.05); (**b**) fracture microstructure and Si elemental mapping of FA- and ALN-impregnated nMBG@CPC after immersion in SBF for 24 h (Marked in the red circle is the relatively strong Si element mapping).

The literature concludes that the compressive strength of trabecular bone ranges from 2 to 48 MPa, generally below 12 MPa [34]. As shown in Figure 4a, the compressive strength of the CPC-only control group was 69.3 ± 4.9 MPa after soaking in SBF for 1 day. The compressive strength of all groups added with nMBG of nMBG@CPC was significantly lower than that of the CPC-only. This phenomenon could be due to the uneven distribution of the nanoparticles of nMBG filler in the CPC matrix. The nM-2.0ALN@CPC group had the lowest compressive strength of 40.4 ± 3.1 MPa due to the aggregation of nMBG led to stress concentration inside the CPC matrix, thereby reducing the strength. This result echoes the working/setting time test. The high affinity of ALN to the calcium ions prolongs the operating time and reduces the compressive strength. Figure 4b shows the fracture surface of each nMBG@CPC composite after soaking in SBF for 1 day. The sheet-like and reef-like crystal structures formed by the CPC reaction are typical morphologies of product hydroxyapatite (HA). Further analysis of the Si mapping confirms that nMBG was clustered and unevenly distributed in the CPC matrix. The presence of different phases in the matrix without proper bonding or interphase assistance may result in poor connectivity of the substrate, which affects the mechanical strength.

#### 2.3.2. FTIR and XRD Analysis

**Figure 5:**
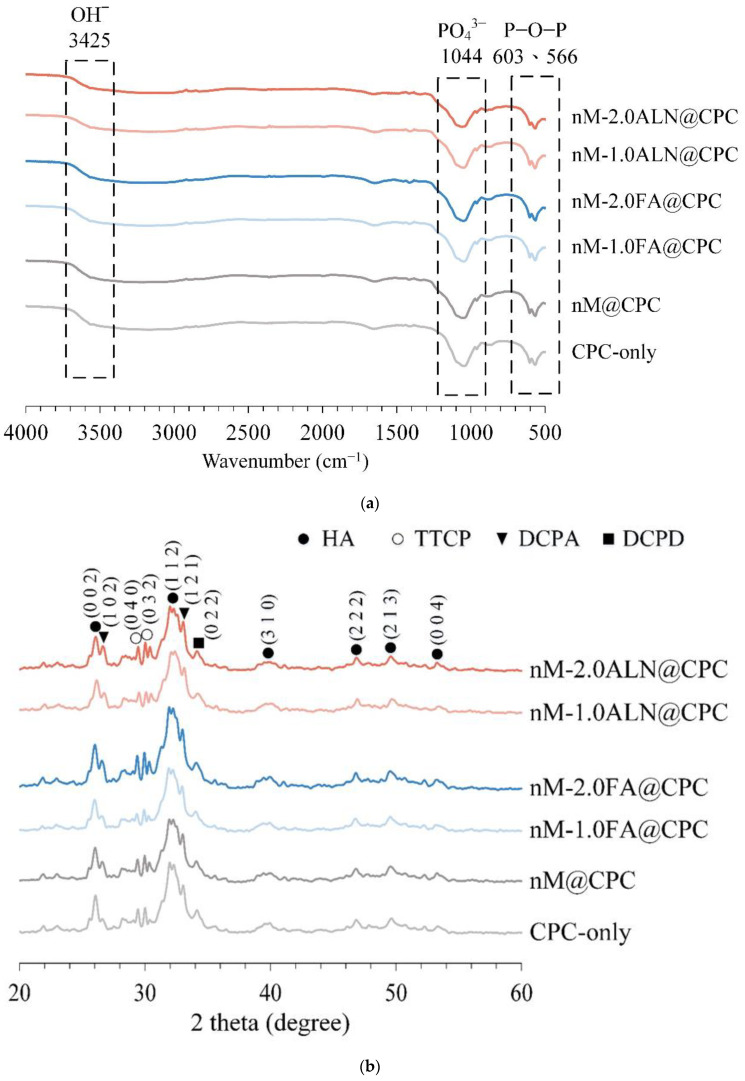
(**a**) Infrared spectra of CPC-only, drug-free nM@CPC, and drug-impregnated nMBG@CPC composites after immersion in SBF for 24 h; (**b**) phasic identification of CPC-only, drug-free nM@CPC, and drug-impregnated nMBG@CPC composites after immersion in SBF for 24 h.

The main product phase of CPC is HA, leading to FTIR spectra (Figure 5a) showing the P-O-P vibrational bands of HA at the absorption of between 566 and 603 cm^−1^. The stretching vibration of the PO_4_^3^-bond of HA was found at 1044 cm^−1^, and the high-frequency vibration of the O−H of HA was evident at 3425 cm^−1^. Compared with CPC-only, the characteristic uptake was not significantly different among the groups, indicating that the addition of drug-impregnated nMBG to CPC did not affect the formation of majority-phase HA.

Figure 5b shows the XRD analysis of each group soaked in SBF for 1 day. Based on comparison with the JCPDS standard file, HA (JCPD 090432) was the main product formed, corresponding to the (0 0 2), (1 1 2), (3 1 0), (2 2 2), (2 1 3), and (0 0 4) planes. The remaining phases were located at 2*θ* of 29.22° and 29.80° with TTCP (0 4 0) and (0 3 2) planes. The (1 0 2) and (1 2 1) planes of DCPA were found at 2*θ* of 26.59° and 32.82°, and the (0 2 2) plane of DCPD was detected at 2*θ* of 34.1°.

ALN is an amino-terminated bisphosphonate molecule with a common P-C-P backbone structure in which P is bonded to a central C atom representing a group of phosphonates. Furthermore, the function of the P-C-P backbone plays a major role in resistance to chemical and enzymatic hydrolysis [35]. Bisphosphonate groups are critical for binding bone minerals and cell-mediated antiresorptive activity of ALN. The mechanism by which ALN inhibits HA crystal growth is caused by the calcium complexation of bidentate chelating bisphosphonates through the interaction of deprotonated oxygen atoms with Ca^2+^ on the HA surface [36]. However, in this study, the functional groups observed by XRD and FTIR analyses mutually confirmed that the dose of ALN at the initial release of nM-2.0ALN@CPC composite did not affect the formation of the apatite phase.

### 2.4. Biocompatibility and Mineralization Activity of CPC@nMBG Composites

#### 2.4.1. L929 Cell Viability

**Figure 6:**
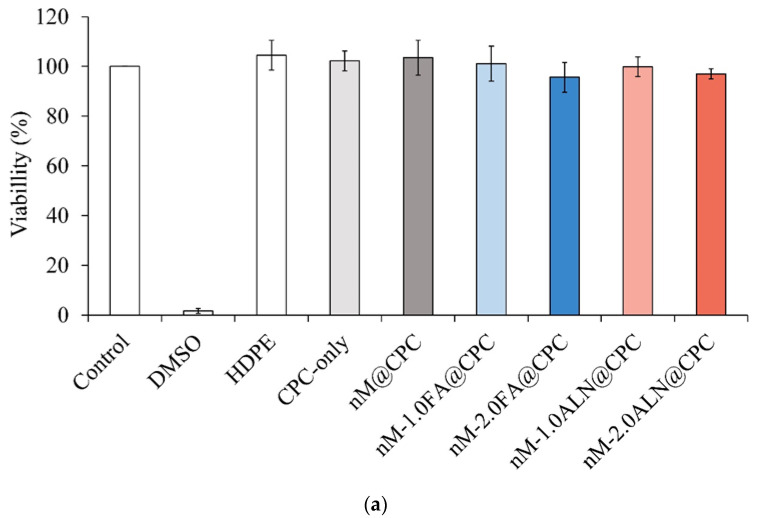

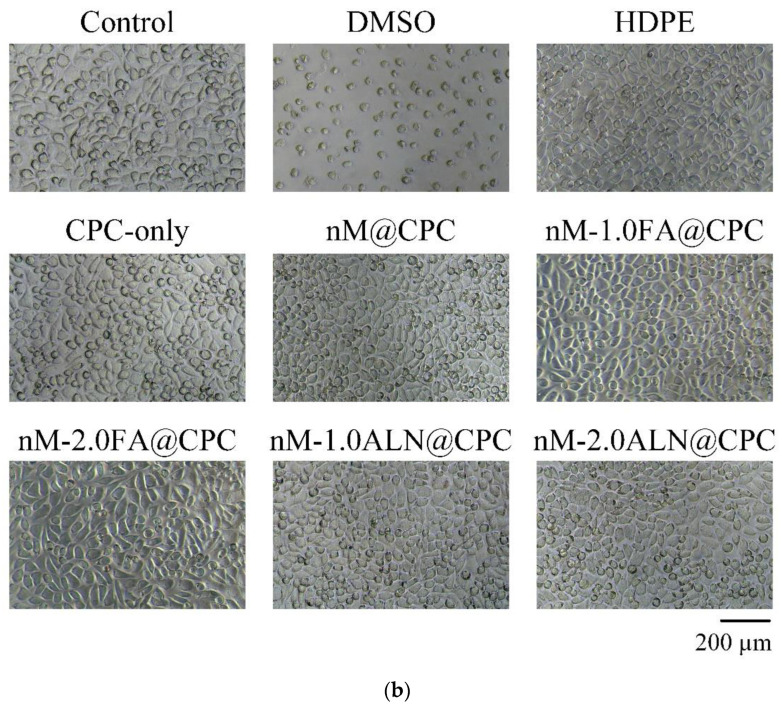
(**a**). Cytotoxicity of drug-impregnated nMBG@CPC in L929 cells cultured for 1 day (*n* = 6); (**b**) morphological observation of drug-impregnated nMBG@CPC in L929 cells cultured for 1 day.

According to ISO 10993-5, an extract of testing material is cytotoxic if the measured cell viability is less than 70% or if a significant change in cell morphology is observed compared to control cells cultured in a normal medium. Figure 6a shows the cytotoxicity test of each group of drug-impregnated nMBG@ CPC composite and L929 cells cultured for 1 day. The survival rate of cells in all groups was higher than 70%, indicating that all composite bone cements were not cytotoxic to L929 cells. Figure 6b shows L929 cells cultured in each extract of nMBG@CPC composite for 1 day. The appearance of control cells was plump and spindle-shaped, indicating that the cells were in good shape. The cell morphology observed in the experimental nMBG@CPC composite group had no significant change from that in the control group, thereby confirming the non-cytotoxicity of the FA and ALN drug release from the nMBG@CPC composite.

#### 2.4.2. Precursor Osteoblasts D1 Mineralization Potential

**Figure 7:**
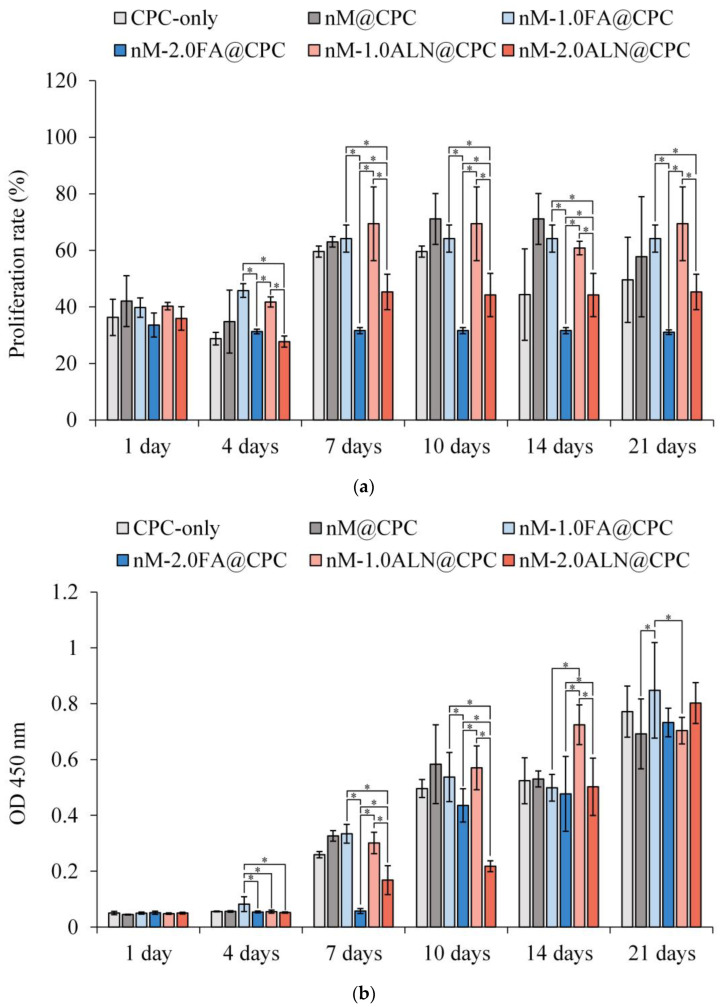

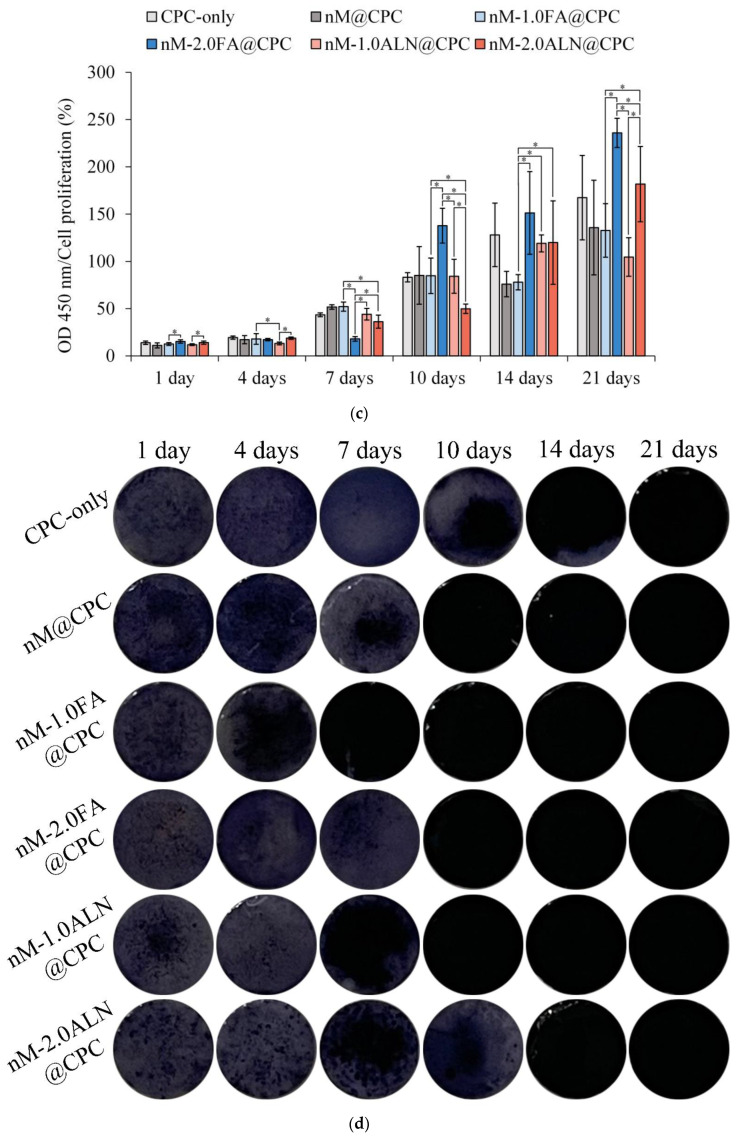

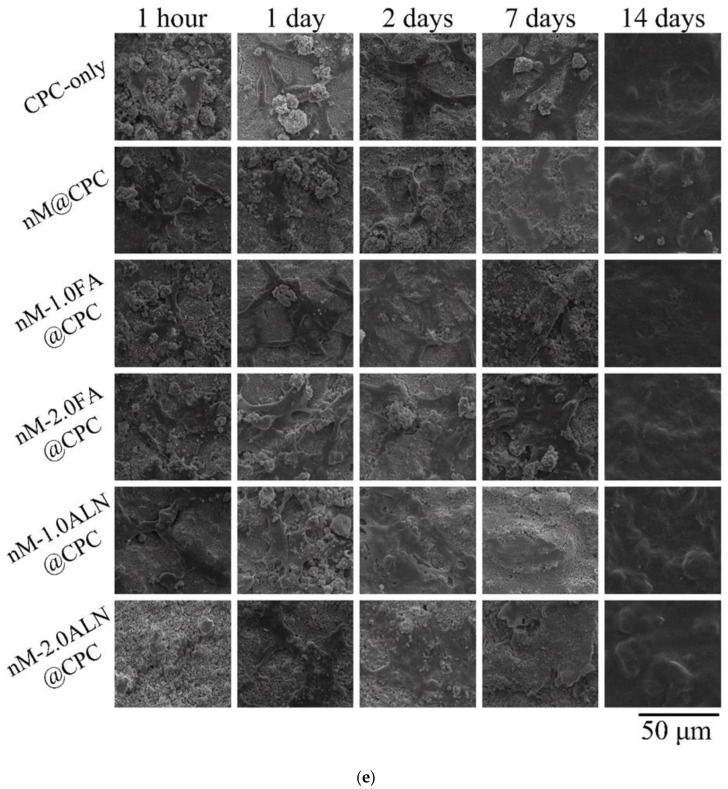
(**a**) Proliferation of D1 cells cultured in drug-impregnated nMBG@CPC for different times up to 21 days (*n* = 3); * indicates significant difference between groups (*p* < 0.05); (**b**) ALP assessment (OD 450 nm) of D1 cells cultured in drug-impregnated nMBG@CPC for different times up to 21 days (OD 450 nm) (*n* = 3); * indicates significant difference between groups (*p* < 0.05); (**c**) Semi-quantitative assessment of ALP (OD 450 nm)/cell proliferation of D1 cells cultured in different groups for up to 21 days (*n* = 3); * indicates significant difference between groups (*p* < 0.05); (**d**) qualitative analysis of ALP staining of D1 cells cultured in each group of MBG composite with CPC for different times; (**e**) morphological observation of D1 cells cultured each group of MBG composite with CPC for different times.

The cell proliferation compared with the blank group is shown in Figure 7a. Overall, the cell viability increased over time and remained stable at day 10, indicating that D1 progenitors entered the mineralization. Based on cell proliferation on the 7th day, the nM-2.0FA@CPC and nM-2.0ALN@CPC groups with high drug concentrations had significantly lower cell viability than the other groups, confirming that high concentrations of FA or ALN were not conducive to the proliferation of D1 progenitor cells. In addition, the cells in the CPC@nM-2.0FA group did not proliferate significantly after 1 day. The FA release concentration of CPC@nM-2.0FA on the 4th day exceeded the release concentration of CPC@nM-1.0 on the 21st day. The drug concentration released by CPC@nM-2.0FA on the 1st day was not conducive to cell proliferation. However, the two drugs had a slight difference in terms of the lower concentration of cell viability at day 21 than in the drug-free nM@CPC control. Overall, FA and ALN still had positive effects on cell proliferation at appropriate low concentrations.

When osteogenic progenitor D1 cells enter the differentiation stage, they will secrete a large amount of ALP to participate in the mineralization; therefore, ALP content was determined to confirm the mineralization activity. In Figure 7b, D1 cells in each group began to secrete ALP in large quantities on the 7th day, indicating that the cells had differentiated, and ALP secretion reached a peak on the 21st day. On the 21st day, the ALP secretion of nM-1.0FA@CPC was slightly higher than that of the other groups, indicating that the appropriate concentration of FA was beneficial to ALP secretion [37]. Figure 7c shows the semi-quantitative analysis of the correlation between ALP and cell viability to accurately analyze the differences in mineralization capacity among groups. The nM-1.0FA@CPC and nM-1.0ALN@CPCALP groups with low drug concentrations showed less ALP secretion than the high-concentration groups. The amount of ALP secreted in the nM-2.0FA@CPC group was significantly higher than that in the other groups. Although a high concentration of FA was not conducive to cell proliferation, it effectively enhanced the mineralization activity of active cells. The comprehensive performance of FA was not inferior to that of the commercially available drug ALN at the same concentration. After confirming ALP production at each time point by staining, the ALP secretion in Figure 7d was similar to that in Figure 7b in all groups at the initial D1 cell culture and was not significantly different after 7 days of cell culture. Figure 7e shows the cell types in each group after D1 cells were contact cultured at different times. In the sample cultured for 1 h, cells in the CPC-only group were spherical, whereas D1 cells in the other groups were all initially attached. After 1 day of culture, most of the D1 cells in each group were evenly attached to the sample surface; on the 14th day, D1 cells were completely attached and covered the sample surface. Overall, impregnating FA or ALN had no direct effect on D1 cell attachment, so nMBG complexed with CPC made the surface rougher than CPC only, which could further promote pseudopodia adhesion, anchoring, and extension of D1 cells.

The method of drug incorporation into CPC slurry during preparation can affect the release behavior of the bone cement matrix [38,39]. The physical and chemical properties of the cement after setting, the additives in the cement matrix, the type of drug, and the environment affect the decay rate after implantation and are thus comprehensive factors that determine the drug release profile. For example, exposure conditions, such as pH, temperature, and medium for the culture of composite bone cement, play a crucial role in drug release kinetics. When CPC powder and liquid are mixed into a slurry, the calcium phosphates undergo an isometric dissolution, precipitation, and phase-transformed process, whereby the entanglement of precipitated apatite crystals is responsible for cement hardening.

Recently, attempts have been made to create various methods and techniques to obtain the application of non-toxic forms of nanoparticles with biocompatibility requirements as drug carriers for modulated drug release [40,41,42,43,44,45,46,47]. Given this, to obtain sustained release and reach efficient concentration, combining nanoparticles with complex shapes as drug carriers with artificial medical devices is the current trend in the treatment of complex indications [42,43]. At the same time, these nanoparticle composites are designed to minimize drug release limitations so that various bone tissue engineering and drug-loading system applications can be developed [44,45,46,47]. The impregnated drugs are released from nanoparticles of nMBG through several processes, besides drug diffusion and MBG erosion, the release of FA and ALN in nMBG@CPC composites is more complex, such as distribution through CPC matrix, release through CPC degradation, dissolution, and diffusion through microchannels present in the CPC matrix grains or formed by erosion [41,43]. In the present study, a bioceramic composite of CPC and bioactive glass was injected into the human body as a paste and set in an environment in the presence of liquid. The injectable composite bone cement of drug-impregnated nMBG@CPC can be used to fill bone voids, for example, after bone resection, regardless of the complexity of the shape and geometry of the void due to its plasticity. In addition, they can be loaded with FA and ALN drugs designed to be delivered to the target site to promote the healing and regeneration of bone tissues for customized osseointegration and bioresorbability. Nonetheless, bioceramics are brittle, so they do not possess the mechanical strength required for orthopedic load-bearing applications [48,49]. ALN is the most commonly used oral nitrogen-containing bisphosphonate for the treatment of osteoporosis, and FA is an emerging therapeutic drug, both of which can inhibit the activity of osteoclasts [18,19]. However, limited reports reveal its response to precursor osteoblasts. From the D1 cell culture experiments in this study, although the two anti-osteoporosis drugs, ALN and FA, cannot effectively promote cell proliferation, they can indeed enhance mineralization.

## 3. Materials and Methods

### 3.1. Materials

The sources of raw materials used in the preparation of CPC bone cement, MBGs, and adjusted hardening solution were as follows: tetracalcium phosphate (Ca_4_P_2_O_9_, TTCP; Realbone Technology Co., Ltd., Kaohsiung, Taiwan), surface-modified dicalcium phosphate anhydrous (sm-DCPA; Realbone Technology Co., Ltd., Kaohsiung, Taiwan), cetyltrimethylammonium bromide (CTAB; Ferak Berlin GmbH, Berlin, Germany), ethyl acetate (Choneye Pure Chemicals, Chennai, India), ammonium (Panreac, Barcelona, Spain), tetraethyl orthosilicate [Si(OC_2_H_5_)_4_, TEOS; ACROS ORGANICS, Geel, Belgium], triethyl phosphate [(C_2_H_5_)_3_PO_4_, TEP; Alfa Aesar, Johnson Matthey Company, Devens, MA, USA], calcium nitrate [Ca(NO_3_)_2_ 4H_2_O, Katayama Chemical Industries Co., Ltd., Osaka, Japan], flufenamic acid (FA; Sigma-Aldrich, St. Louis, MO, USA), and alendronate acid (ALN; Sigma-Aldrich, St. Louis, MO, USA).

### 3.2. Preparations of CPC-Only, nMBG, nMBG-FA, nMBG-ALN, and nMBG@CPC Composites

#### 3.2.1. CPC-Only

Our previous study revealed the process of sintering, pulverization, and powder control of TTCP and the surface modification of sm-DCPA [20]. The CPC powder has a Ca/P composition of 1.67 based on a mixed powder of 16.6 g of TTCP and 12.4 g of sm-DCPA, and the mean particle distributions of TTCP and sm-DCPA used for CPC-only were 12.6 and 2.1 μm, respectively. The concentration of the hardening solution prepared by dissolving NaH_2_PO_4_ was 0.67M, and the final pH value was adjusted to 6.02. It is important to note that pH will change due to storage conditions and should therefore be monitored before use.

#### 3.2.2. nMBG, nMBG-FA, and nMBG-ALN

In this experiment, a base-catalyzed method was used to prepare nMBG with mesoporous BG nanoparticles through sol–gel method. In brief, 0.7 g of CTAB surfactant was dissolved in 33 mL of ddH_2_O, added in sequence with 10 mL of ethyl acetate-emulsified oil phase, 7 mL of ammonia water (concentration 3 M), 3.6 mL of TEOS, 0.36 mL of TEP, and 2.3 g calcium nitrate, and stirred for 4 h prior to filtration. The precipitate was washed three times with ddH_2_O and ethanol, dried at 60 °C, sintered to 650 °C at 2 °C/min, and calcined for 3 h to obtain nMBG.

Drug-impregnated nMBG samples were prepared. FA was dissolved in alcohol at between 1.0 and 2.0 mg/mL, and ALN was dissolved in ddH_2_O. The nMBG was immersed in a large amount of 1 g/50 mL drug-containing solution for 1 day, air-filtered, and dried to obtain the drug-impregnated nanoparticles of nMBG (designated as nM-1.0FA, nM-2.0FA, nM-1.0ALN, and nM-2.0ALN).

#### 3.2.3. Drug-Impregnated nMBG Composite CPC

The proper additive of 5 wt.% nMBG in the CPC matrix was investigated based on our previous study and the literature [9,21]. The CPC-only composite was set as the control group, and nMBG@CPC composites consisting of CPC powder mixed with drug-free 5 wt.% nMBG were used for comparison. The 5 wt.% nMBG composites were impregnated with different concentrations of FA and ALN (designated as nM-1.0FA@CPC, nM-2.0FA@CPC, nM-1.0ALN@CPC, and nM-2.0ALN@CPC). The composite slurries were prepared by mixing the above MBG/CPC powder with 0.67 M phosphate hardening solution at a powder-to-liquid ratio of 1.0 g to 437.5 μL. The slurry was well mixed for 1 min and then pressed at 0.7 MPa (100 psi) into a cylindrical sample with a height × diameter of 12 × 6 mm. The sample was left to set for 3 min before demolding. Physical and chemical properties were then evaluated.

### 3.3. Physiochemical Properties

#### 3.3.1. Nanoparticle Identification by Transmission Electron Microscopy (TEM)

nMBG ultrasonically dispersed in alcohol was dropped on a carbon-coated copper grid. After drying, morphology changes in nMBG before and after drug impregnation were observed with TEM (JEM-2100F, JEOL, Tokyo, Japan).

#### 3.3.2. Drug Release Measurement by Ultraviolet-Visible (UV-Vis) Spectrometer

About 0.8 g of nMBG sample was soaked in 4 mL of simulation body fluid (SBF) buffered with TRIS. Optical density at 288 nm (OD_288_) was analyzed in groups containing FA at different time points by using a UV-Vis spectrometer (UV1800, Shimadzu, Kyoto, Japan) to measure changes in drug release.

#### 3.3.3. Spectra of Fourier Transform Infrared Spectroscopy–Attenuated Total Reflectance (FTIR-ATR)

After soaking in SBF for 24 h, the drug-impregnated nMBG@CPC composites were ground into powder, mixed with dry KBr powder at a ratio of 1/100 (g/g), and pressed into translucent circles with a diameter of 12 mm. Recorded spectra using the ATR mode of the FTIR spectrometer (Nicolet 6700, Thermo Fisher Scientific, Waltham, MA, USA).

#### 3.3.4. X-ray Diffraction (XRD) Patterns

XRD was used to analyze the effect of drug-free nMBG and drug-impregnated nMBG composite CPCs on crystallization. The composite bone cement was reacted for 24 h, immediately ground into powder, and analyzed by XRD diffractometer (XRD-6000, Shimadzu, Kyoto, Japan). Diffraction conditions included Ni-filtered Cu target Kα, 30 kV, 20 mA, scan angle within the range of 20–60°, and a 2θ scan rate of 2°/min. According to the JCPDS database, the obtained XRD patterns were compared with standard diffraction peaks.

#### 3.3.5. Working Time and Setting Time

Working time is defined as the time when bone cement is mixed into a slurry and gradually hardens without mutual adhesion between the pastes in the slurry. The setting time of the CPC slurry showed certain strength after initial hardening. According to the dental phosphoric acid standard ISO 9917-1, measuring the setting time was using a 400 g Gill-more needle to press vertically on the surface of the sample under the conditions of 37 °C and 60–70% humidity and recorded the initial setting time until no indentation was visible on the surface.

#### 3.3.6. Injectability and Disintegration Resistance

Syringability and disintegration were tested by mixing the slurry for 1 min and transferring it to a 5 mL needle-free syringe within 3 min. The immersion of the composite bone cement was observed. The lack of further disintegration after 1 day of immersion indicates resistance to disintegration.

#### 3.3.7. Sample Compression and Fracture Observation

Testing was performed following ASTM F451-16. The diameter and height of the composite samples immersed in 1 g/10 mL TRIS were 6 mm × 12 mm. The samples were soaked at 37 °C for 1 day, collected, wet pressed by wiping the surface at a crosshead speed of 1 mm/min, and analyzed using a universal testing machine (HT-2402, Hung Ta, Taichung, Taiwan). The fractured microstructure of the composite bone cement after compression and the Si mapping distribution of nMBG in the CPC matrix was observed using a scanning electron microscope (SEM; S-3400N, Hitachi, Tokyo, Japan) equipped with an energy dispersive spectrometer (EDS).

### 3.4. In Vitro Cytotoxicity

Neonatal mouse fibroblast L929 cell line was purchased from the National Institutes of Health, Miaoli, Taiwan for cytotoxicity assays according to ISO 10993-5:2009. Cell culture followed protocol in Minimal Essential Medium alpha medium (Gibco, Thermo Fisher Scientific Inc., Waltham, MA, USA) supplemented with 10% horse serum. The medium was changed every 2 days during the experiment. The samples were sterilized by autoclaving at 121 °C and 1.05 kg/cm^2^ (15–20 psi; TOMIN, TM-328, Taipei, Taiwan). The ratio of extract to medium for composite samples was 1 g/5 mL. Cells were cultured in the extracted medium for cytotoxicity assays and incubated for 24 h in a 37 °C incubator. The negative control used was high-density polyethylene (HDPE), and the positive control was 15 vol.% dimethylsulfoxide (DMSO; Sigma-Aldrich, St. Louis, MO, USA) for comparison with extracts from composites to ensure the validity of the test.

For quantitative cytotoxicity assay, transferred 100 μL of the suspension containing L929 1 × 10^4^ cells into a 96-well microplate and incubated for 24 h at 37 °C. The medium was then removed and re-introduced with 100 µL of the sample extract for an additional 24 h, followed by a 4 h extension of the reaction mixture with 50 µL of the XTT Cell Proliferation Assay Kit (Bioindustry, Kibbutz Beit Haemek, Israel). The samples were processed with an ELISA reader (SPECTROstar Nano, BMG LABTECH, Offenburg, Germany) to measure OD_490_ absorbance, which is directly proportional to cell viability. As for the qualitative test, after culturing L929 cells in the extract (100 μL) of the composite for 24 h, the cell morphology was observed under an inverted microscope (IVM-3AFL, SAGE VISION Co., Ltd., New Taipei City, Taiwan).

### 3.5. Enhanced D1 Cell Proliferation and Osteogenic Differentiation

#### 3.5.1. Cell Attachment and Proliferation

For further in vitro testing, progenitor cells from the mouse precursor osteoblast line D1 were cultured on the nMBG@CPC composite matrix to observe various cellular activities and cell mineralization. The D1 cell culture medium used was Dulbecco’s modified Eagle medium (Thermo Fisher, Waltham, USA) supplemented with 10% fetal bovine serum. Disc composite samples with dimensions of 6 mm × 3 mm were seeded in 48-well plates and cultured in contact with D1 cells at a cell density of 1 × 10^5^ cells/well. Samples were incubated for 1, 4, 7, 10, 14, and 21 days. The samples were incubated for 1, 4, 7, 10, 14, and 21 days. The cell culture medium was incubated with the Alamar Blue Proliferation Assay Kit (Bio-Rad, Hercules, CA, USA), and the samples were mixed for an additional 4 h. The optical density at OD_570_ and OD_595_ was measured using an ELISA reader to quantify cell viability and proliferation. After fixation, the adhesion and morphology of cells on the composite samples at different culture times were observed by SEM.

#### 3.5.2. Semi-Quantitative Mineralization of Alkaline Phosphatase (ALP) Activity and Staining

ALP, an early marker of osteogenesis, was quantified by dissolving a p-Nitrophenyl phosphate tablet and a TRIS tablet in 20 mL of sterile water and mixing them well. After the cells had been cultured for a set time, the cells were washed with phosphate-buffered saline, and the quantified assay was added to the prepared medium solution. Then, D1 cells were incubated for an additional 30 min, and absorbance at OD_405_ was measured using an ELISA reader. The amount of ALP secreted by the cells was directly proportional to the absorbance obtained at OD_405_.

ALP staining assay medium was prepared by dissolving 1% Alcian blue (Sigma-Aldrich, St. Louis, MO, USA) in citrate buffer (pH 5.4) containing 45% acetone and 10% methanol fixative. After incubation, the samples were fixed with 4% paraformaldehyde, washed with deionized water, and added to the prepared ALP staining assay for staining.

### 3.6. Statistical Analysis

Analysis of variance (ANOVA) and two-sample *t*-test for differences in composite bone cement was performed using IBM SPSS Statistics ver. 20 (IBM, BY, New York, NY, USA). ANOVA was conducted to estimate the significance of the difference in means for two different variables that were significantly different from each other when *p* < 0.05.

## 4. Conclusions

The drug release mechanism is atypical Fick diffusion, which confirms that the drug is indeed impregnated into nMBG to achieve the effect of sustained release. The results confirmed that the incorporation of drug-impregnated nMBG@CPC composites did not affect the formation of the HA product phase. The working time of the nMBG@CPC impregnated with ALN was slightly higher than that of the other nMBG@CPC composites. Meanwhile, the working/setting time of the nMBG@CPC composites was shortened compared with CPC-only. The compressive strength of the nMBG@CPC composites was reduced after the addition of nMBG but still met the clinical application requirements. In addition, all drug-impregnated nMBG@CPC composites revealed good injectability, and the injected sample had good disintegration resistance after soaking for 1 day. None of the groups showed toxicity. Cell contact culture showed that the group with higher drug impregnation was not conducive to the proliferation and mineralization of precursor osteoblast D1 cells. However, in comparing ALP secretion versus cell viability at 21 days, higher concentrations of FA and ALN enhanced the mineralization capacity of D1 cells. Although related trials of osteoclast inhibition are needed in the future, according to the current results, FA or ALN drug-impregnated nMBG@CPC composite can be a suitable bone cement for filling and repairing osteoporosis-related bone defects.

## Data Availability

The data presented in this study are available on request from the corresponding author.
